# Perceptions of intelligence & sentience shape children’s interactions with robot reading companions

**DOI:** 10.1038/s41598-023-32104-7

**Published:** 2023-05-05

**Authors:** Nathan Caruana, Ryssa Moffat, Aitor Miguel-Blanco, Emily S. Cross

**Affiliations:** 1 School of Psychological Sciences, Macquarie University, Level 3, 16 University Ave, Sydney, NSW 2109 Australia; 2 Centre for Elite Performance, Expertise and Training, Macquarie University, Sydney, Australia; 3 Institute of Neuroscience and Psychology, University of Glasgow, Glasgow, UK; 4 MARCS Institute for Brain, Behaviour and Development, University of Western Sydney, Sydney, Australia; 5 Department of Humanities, Social & Political Sciences (D-GESS) and the Department of Health Sciences and Technology (D-HEST), ETH Zurich, Zurich, Switzerland

**Keywords:** Human behaviour, Psychology

## Abstract

The potential for robots to support education is being increasingly studied and rapidly realised. However, most research evaluating education robots has neglected to examine the fundamental features that make them more or less effective, given the needs and expectations of learners. This study explored how children’s perceptions, expectations and experiences are shaped by aesthetic and functional features during interactions with different robot ‘reading buddies’. We collected a range of quantitative and qualitative measures of subjective experience before and after children read a book with one of three different robots. An inductive thematic analysis revealed that robots have the potential offer children an engaging and non-judgemental social context to promote reading engagement. This was supported by children’s perceptions of robots as being intelligent enough to read, listen and comprehend the story, particularly when they had the capacity to talk. A key challenge in the use of robots for this purpose was the unpredictable nature of robot behaviour, which remains difficult to perfectly control and time using either human operators or autonomous algorithms. Consequently, some children found the robots’ responses distracting. We provide recommendations for future research seeking to position seemingly sentient and intelligent robots as an assistive tool within and beyond education settings.

## Introduction

The past 15 years have seen growing confidence and interest in the potential for social robots to support children’s education, with a large body of research already evaluating the use of robots in various education support roles^1^. Initial reports of success have been attributed to the physical embodiment and social presence of robots, which may promote learning engagement^2^. However, very little research has examined precisely which aesthetic and functional features make robots more beneficial than other forms of technology and media.

Closely examining *why* and *how* particular robots can facilitate learning in different contexts is important given the high degree of variability across the types of robots used in education, the roles they play during learning, and the type of learning they support^2^. As such, the field now needs a systematic interrogation of the features that help optimally position robots to promote learning. Ideally, this foundational research should be conducted *before* robots are selected for education interventions. This will not only inform which robots are likely to be the most effective for the skills and learners targeted, but can mitigate the use of unnecessarily expensive, complex, or poorly suited robots.

A recent systematic review by Papakostas and colleagues on the use of robots in special education highlights this multiplicity problem across what is a relatively nascent field of enquiry^2^. Whilst outcomes across studies were mostly encouraging, researchers have used very different robots to target different skills, and with children who have a variety of different special education needs. Further, most studies were underpowered, underrepresented female learners, and lacked critical control conditions. These studies also neglected to examine children’s perceptions and experiences of the robots. This is striking given that social neuroscience inspired human—robot interaction research has repeatedly evidenced the powerful influence of human perceptions, beliefs and expectations on interactions with both virtual and robotic artificial agents^3–7^ (also see Hortensius & Cross^8^ for a relevant review). Whilst understanding how these perceptions shape interactions with artificial agents is a highly complex and challenging endeavor, it should be a central focus as we develop, deploy and evaluate robot assistants^9^. This is especially critical when considering how best to design robots to enhance the early education of children.

With the current study, our aim was to begin addressing this challenge by examining children’s first impressions, expectations and experiences while engaging with three different robots (NAO, MiRo, Cozmo) that varied in appearance and function. We specifically examined these questions in the context of a reading activity to probe the extent to which these robots might be ‘fit-for-purpose’ as a tool to support the socio-emotional context and engagement of children and the features that are likely to make them more or less so.

### Robots for reading

Some studies have already begun to examine the extent to which reading robots can be used to support children’s learning. However, prior work has mostly focused on using ‘storyteller’ robots in group settings where the robot is unable to support a one-on-one interaction with a child^10,11^. Surprisingly, little work has been done to examine whether social robots can support children’s reading in one-on-one interactions. This is likely due to the complex and dynamic nature of evidence-based reading instruction which currently outstrips the capabilities of most robots. For instance, phonics training requires instructors to provide timely and sensitive feedback on the pronunciations of words read aloud^12,13^. Current speech recognition systems are not able to reliably and rapidly recognise words that are mispronounced. Despite this, robots may yet be able to promote reading engagement and practice as a supplement to targeted reading instruction provided by human educators. A recent study conducted by Michaelis and Mutlu^14^ provided some insight into the potential of such interventions. Here, children who engaged in a guided reading intervention with an in-home social robot, *Minnie*, reported increased motivation and improved reading comprehension. The authors suggest that social robots can support reading in several ways, including by simulating social interactions that promote comprehension and opportunities for ‘connection-making’, as well as by modelling effective reading habits. However, as we explore in a recent review, social robots might also be particularly useful in supporting reading in children who experience reading difficulty – and associated reading anxiety – by providing a social context for reading that is both engaging and unintimidating^15^.

It is now well-established that poor reading and poor mental health outcomes are related^16,17^. Specifically, it is likely that reading-related anxiety hinders reading gains due to increased reading avoidance. This is consistent with findings that children with below-average reading score higher on specific measures of reading anxiety than children with more established reading ability^18^. Furthermore, interventions that simultaneously address children’s reading difficulty and anxiety through evidence-based reading instruction yield significant improvements in both reading and mental health outcomes^19^. The success of these interventions is, in part, attributed to their highly individualised approach. They are, however, time intensive and expensive. Despite this, little work is being done to explore additional tools and technologies that can be deployed in the classroom or home to supplement or reinforce children’s reading engagement – and as a result, learning – beyond direct instruction interventions. One avenue is the use of technology to encourage children to practice their reading between teacher-led instruction sessions. This is critical given that the frequency of reading engagement is positively associated with reading skill acquisition^20^. Additional interventions which promote practice, without direct instruction or feedback have the potential to increase confidence and thus mitigate anxious avoidance during reading instruction opportunities in classroom settings.

In 1999, the Reading Education Assistance Dogs (READ) program was launched in the United States, which has seen sustained global reach. The program involves children reading to dogs, and is founded on the basic principle that dogs can increase motivation, relaxation and confidence by establishing a non-judgemental social context for reading^21^. The program also assumes that reading anxiety and avoidance play a part in maintaining reading difficulties. A systematic review of 48 papers evaluating this approach presents a relatively consistent account of dogs having a positive impact on children’s reading engagement, anxious arousal and reading attainment outcomes. However, the quality of this evidence is underwhelming, with the vast majority of outputs falling within the lowest category (Level 5) of the Oxford Centre for Evidence Based Medicine (OCEBM) levels of quality criteria^21^. Assuming that the benefit of reading dogs rests on their engaging and non-judgemental social presence, social robots may provide a much more practical alternative. Unlike dogs, robots offer a higher degree of predictability and user control, which can mitigate the possibility of distracting children when reading. Robots can also be designed to engage, respond, and interact in a way that is relevant to the story being read. This is important given growing evidence that greater learning gains are observed in interventions where robots display responsive and personalised social behaviour^1^. Robots are also more scalable and practical for deployment in the classroom and home since they require minimal ongoing care/maintenance. Further, many commercially-available robots can be easily programmed and adapted to serve as a reading buddy. Finally, robots may present a more accessible and equitable solution than reading dogs. Whilst the cost of social robots ranges widely, many are affordable; some costing less than a video game console or personal computer.

To this end, the current study aimed to explore the robot features that lead to both positive and negative expectations and experiences when children interact with a robotic reading companion. To do this, our study employed in-depth qualitative measures to capture children’s first impressions, and first-hand experiences of interacting with three different social robots during a reading task. We implemented a qualitative inductive thematic analysis following an interview which explored children’s experiences which was supplemented and contextualised by quantifiable subjective measures collected before, during and after children were exposed to the different robots. Close attention was paid to how children perceived the robot’s fundamental social capabilities – including how intelligent, attentive and kind they believed the robot was. We also explored the physical robot features (e.g., aesthetics and function) that gave rise to these initial impressions. These findings will directly inform our understanding of the aspects of social robots most likely to promote reading engagement in children.

## Results

Children’s perspectives were sampled in several phases to characterise their first impressions and expectations of the different robots included in the study (NAO, Cozmo, and MiRo), as well as their experiences during a sustained interaction with a robot of their choice (Nao, n = 15; Cozmo, n = 11; Miro, n = 3). In Phase 1, we sampled children’s first impressions of each robot. Children were asked to rank the three robots according to their preference and rate each on their perceived ‘Intelligence’ and ‘Friendliness’. Children provided these ranks and ratings twice; upon entering the lab and viewing the inactive robots, and again after watching a video of each robot performing a standardised action. In Phase 2, children were asked to select one robot to read with. During the reading activity, children read aloud to the robot, and the robot responded appropriately to several pre-defined plot points during the story using a Wizard-of-Oz approach. After the reading task, in Phase 3, children participated in a semi-structured interview where they provided several subjective ratings of their robot reading companion and their reading experience. Children were also asked several open-ended questions about their experience taking part in the study, their chosen robot, and the features they would expect of an ideal robot reading companion. Responses were analysed using a reflexive and inductive thematic analysis (see Methods section for full procedural and analysis details).

## First impressions and expectations

### Robot preferences

**Figure 1:**
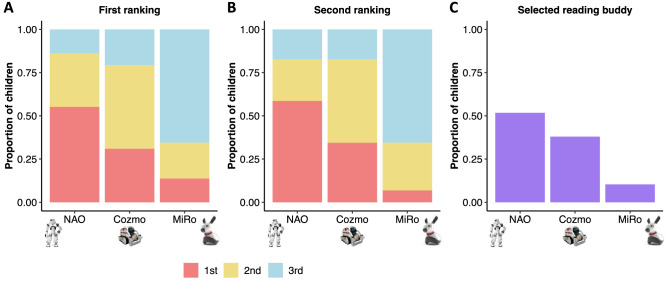
Bar graphs depict the proportion of children who ranked each robot in each place, i.e., 1st, 2nd, 3rd, (**A**) immediately upon viewing the inactive robots and (**B**) after viewing the short video demonstrations of the robots in action. Panel (**C**) summarizes the proportion of children who selected each robot as their reading buddy.

Children ranked the robots in order of preference immediately upon viewing the inactive robots (Fig. 1A), and again after watching short videos depicting each of the robots in action (Fig. 1B). Children were then provided with a final opportunity to select a robot to read with (Fig. 1C). On final selection, most children preferred NAO (n = 15, 51.72%), followed by Cozmo (n = 11, 37.93%) and then MiRo (n = 3, 10.34%). An exploratory analysis revealed a significant effect of robot on the probability of being selected (χ2 (2) = 7.72, *p* = 0.021). Post-hoc tests revealed that children were significantly more likely to select NAO over MiRo (χ2 (2) = 8.00, *p*_*FDR*_ = 0.014), and Cozmo over MiRo (χ2 (2) = 4.57, *p*_*FDR*_ = 0.049). No significant differences were noted in post-hoc comparisons between NAO and Cozmo (χ2 (2) = 0.62, *p*_*FDR*_ = 0.443).

Taken together, this suggests that the zoomorphic MiRo robot was markedly less popular in this context, perhaps reflecting a reduced perceived capacity to engage in a reading task. It is of note that three of the four children who initially ranked MiRo first changed their preferences upon viewing the videos. By comparison, 6/16 children who initially ranked NAO first, and 5/9 children who ranked Cozmo first changed their preferences, after watching the video. Whilst the overall preference pattern remained stable across ranking opportunities, 47% of children changed their first preferences after watching the videos, suggesting that both aesthetic form *and* function play a role in shaping first impressions and expectations.

### Intelligence and friendliness ratings

**Figure 2:**
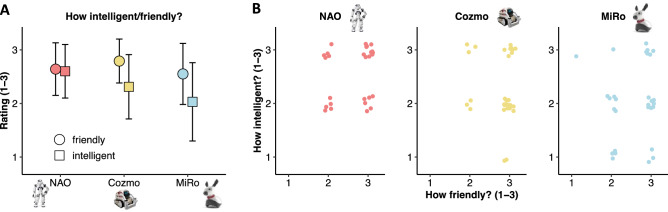
(**A**) Mean ratings for ‘How intelligent’ and ‘How friendly’ children found each robot to be after viewing demonstration videos. (**B**) Scatterplot summarizing the relationship between individual ratings of Intelligence and Friendliness for each robot.

After viewing the demonstration videos, children rated how ‘intelligent’ and ‘friendly’ they thought each of the three robots were on a 3-point scale (1–3; see Methods and Fig. 2A). We found evidence for a significant effect of robot on intelligence ratings (*χ2* (4) = 13.12, *p* = 0.011). Post-hoc tests revealed that NAO (*M* = 2.61, *SD* = 0.50) outperformed MiRo (*M* = 2.03, *SD* = 0.73; *χ2* (2) = 10.60, *p*_*FDR*_ = 0.015), but not Cozmo (*M* = 2.31, *SD* = 0.60; *χ2* (2) = 4.20, *p*_*FDR*_ = 0.184), with no significant differences noted between Cozmo and MiRo (*χ2* (2) = 3.38, *p*_*FDR*_ = 0.184).

We found no evidence for significant robot effects on ratings of friendliness (NAO: *M* = 2.64, *SD* = 0.49; Cozmo: *M* = 2.79, *SD* = 0.41; MiRo: *M* = 2.55, *SD* = 0.57; *χ2* (4) = 4.57, *p* = *0.335*).

## Reading experiences

### State anxiety immediately before and after reading with robot

**Figure 3:**
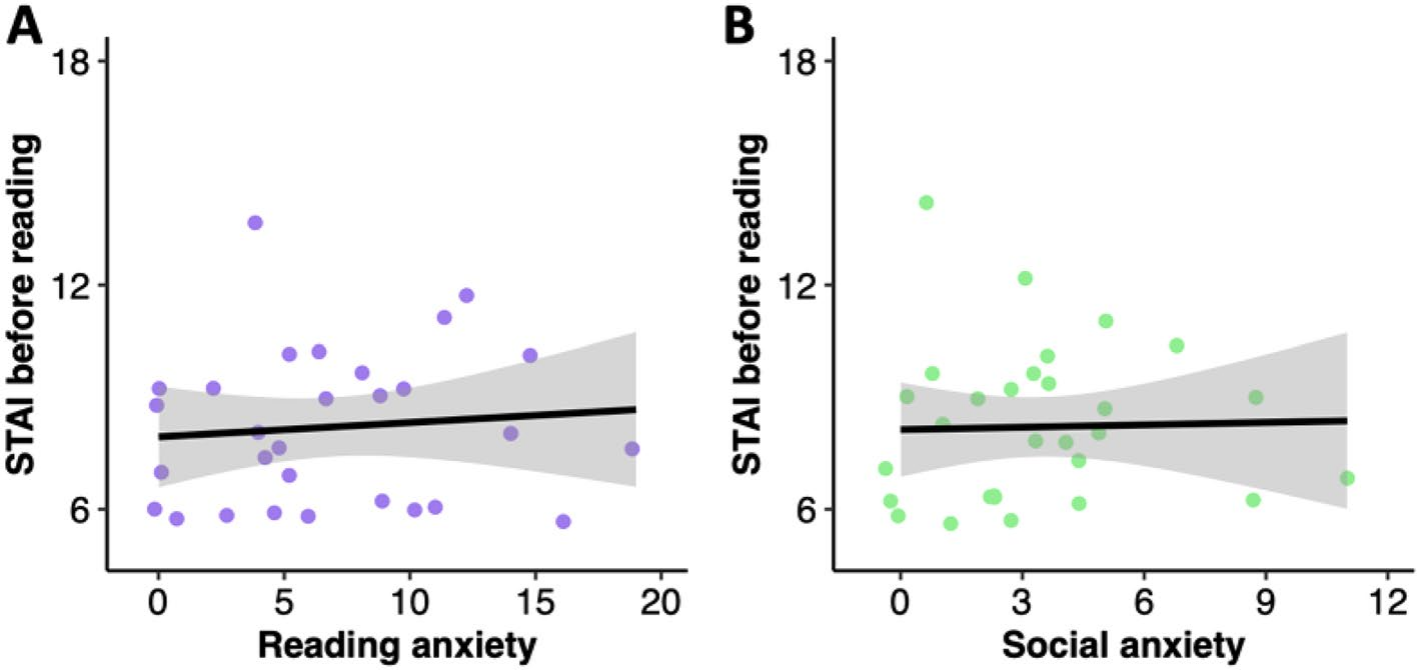
Scatterplot depicting the association between pre-reading state anxiety and (**A**) MORAT Reading Anxiety; and (**B**) SPENCE social anxiety.

We collected state anxiety measures from children immediately before (M = 2.21; SD = 2.08) and after (M = 0.52; SD = 0.87) reading with the robot to explore whether there were any children who experienced marked anxiety immediately before or after reading with the robot. This was not the case. We also ran two exploratory Spearman correlation tests to determine whether reading anxiety and social anxiety traits were associated with elevated state anxiety immediately before reading. We found no evidence for a significant association between our state anxiety measure and scores on either the MORAT measure of reading anxiety (ρ = 0.13, p = 0.659) or SPENCE social anxiety subscale (ρ = 0.19, p = 0.659; see Fig. 3).

### Subjective ratings of selected reading robot

**Figure 4:**
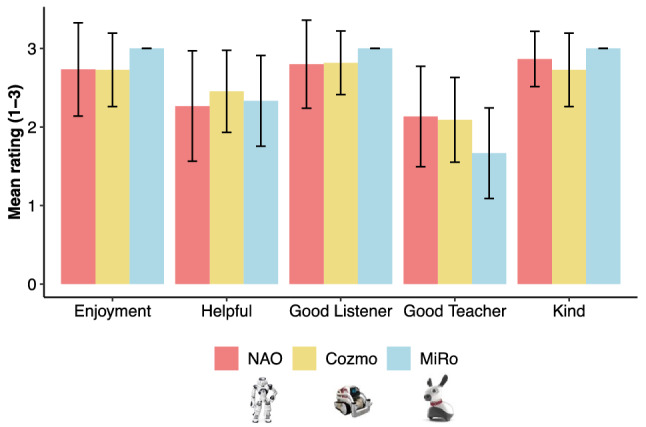
Subjective ratings of Enjoyment as well as the extent to which the robot was, ‘Helpful’ a ‘Good Listener’, a ‘Good Teacher’, and ‘Kind’. *Note*. Here children rated their experience with their selected robot (Nao, n = 15; Cozmo, n = 11; Miro, n = 3).

After reading with their selected robot, children were asked to indicate whether they enjoyed reading with the robot, and whether it was helpful. Almost all children agreed with both statements (Fig. 4). However, three children who selected NAO indicated that the robot was *not* helpful. Two of these children commented that NAO’s reactions were unpredictable and distracting: ‘Interrupted me [by] laughing loudly’ [P101, F, 12yrs] and ‘All he does is listen and give random reactions, not helpful because out of nowhere he distracts you’ [P108, M, 12yrs]. The third child explained that NAO was not helpful because it did not offer corrective feedback [P117, M, 10.5yrs]. Two children who indicated that they did *not* enjoy reading with their selected robot, also selected NAO. One (same as above) attributed this to the robot’s distracting behavior.

To obtain further information about children’s perceptions of robots during the reading activity, they were asked to rate how much they enjoyed reading with their selected robot (Nao, n = 15; Cozmo, n = 11; Miro, n = 3), and the extent to which the robot was, ‘Helpful’ a ‘Good Listener’, a ‘Good Teacher’, and ‘Kind’ using the same visual 3-point rating scale (see Fig. 4). Subjective ratings revealed that most children enjoyed reading with their selected robot and felt that the robot was both kind and attentive (i.e., a ‘Good listener’). This was true, despite children having mixed perceptions about the robots’ capacity to directly help or teach them while reading. An exploratory analysis revealed no evidence for an effect of robot on any of these ratings (all ps > 0.458; see accompanying R Markdown for full analysis).

### Preferences for reading with robots vs alone or with teacher

**Figure 5:**
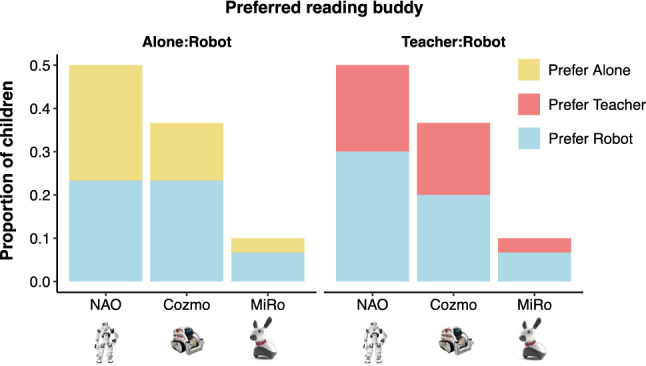
Proportion of children who prefer reading to a robot than on their own or with a teacher, by selected robot.

Finally, children were asked whether they preferred reading with their selected robot over reading alone or with their school teacher. Overall, children presented mixed preferences, with just over half the children preferring to read with the robot over reading alone or reading with their teacher (Fig. 5, Table 1). An exploratory analysis did not offer any evidence for robot-specific effects on either the preference to read alone (*χ2 (2)* = *0.92, p* = *0.632*) or with a teacher (*χ2 (2)* = *0.17, p* = *0.920*) over reading with a robot companion.

**Table 1:** Percentage of children who preferred reading with a robot than alone or with a teacher.

	Alone ~ Robot		Teacher ~ Robot	
	Alone	Robot	Teacher	Robot
NAO	27.59	24.14	20.69	31.03
Cozmo	13.79	24.14	17.24	20.69
MiRO	3.45	6.90	3.45	6.90
Sum	44.83	55.18	41.38	58.62

## Thematic analysis of reading with robot experience

**Figure 6:**
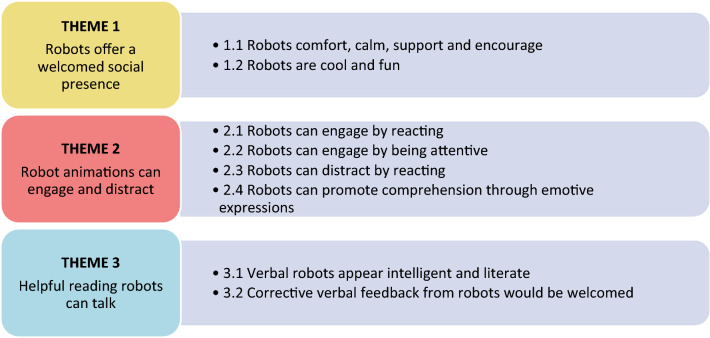
Summary of themes and sub-themes.

Our thematic analyses of interviews with children identified three key themes relating to children’s perceptions and expectations of robot reading companions. Figure 6 summarises these themes, as well as several corresponding sub-themes which are explored in more detail in Supplementary Material 1, which includes all relevant quotes organised by sub-theme, participant, and robot. Quotes are accompanied by the participants anonymised ID, age (years), selected robot and their MORAT-P score, to demonstrate the diversity of evidence sources (in the format: [ID, robot, age, MORAT]. Supplementary Material 1 also includes a table that offers a comprehensive summary of evidence for each of the sub-themes, again organised by selected robot.

## Discussion

The current study aimed to comprehensively examine children’s perceptions and expectations towards three very different robots (NAO, Cozmo, MiRo) to identify features that make a robot well-suited to promote children’s reading engagement. We employed a mixed methods approach in which children shared their impressions of the different robots at three different time points: first, based on the robots’ appearance alone, then upon observing the robots in action, and finally after reading with a robot of their choice. This was followed by in-depth interviews with each child, which we then examined using a reflexive thematic analysis approach.

We found that most children were drawn to NAO, the humanoid robot, upon initially seeing the inactive robots, and after viewing videos of the robots performing dynamic actions. This is consistent with recent findings in which young children were found to prefer NAO over more machine-like and human-like robots^22^. Whilst preferences for the three robots remained consistent overall, almost half the sample changed their preferences after viewing the videos, suggesting that children’s early impressions and preferences for robots are informed by both aesthetic form and function^9,23^.

Whilst all robots received consistently high rating for friendliness, children provided significantly higher intelligence ratings for NAO over MiRo, which may indicate that children perceive this humanoid robot to be intelligent enough to engage in the reading activity, but also friendly enough to mitigate any fear of negative evaluation if the child were to make reading errors. Importantly, subsequent thematic analyses revealed that whilst this was certainly true of some children who read with NAO, it was not unique to humanoid robots, and was also experienced with the machine-like (Cozmo) and zoomorphic (MiRo) robots. Overwhelmingly, children reported positive experiences with their selected robot reading companion, irrespective of the robot chosen. For instance, whilst very few children selected MiRo (n = 3), each of these children reliably reported enjoying the experience and perceived MiRo to be highly attentive and kind (see Fig. 6). Whilst this apparent reliability may be an artifact of the small number of children who selected MiRo – these experiences may suggest that robotic ‘pets’ such as MiRo may offer similar benefits to those previously reported with reading assistance dogs^21^.

Our thematic analyses identified three key themes that provide valuable insight into children’s perspectives towards robots as reading companions, the features that best position robots to fulfill this role, as well as those that present challenges. Children expressed that: (1) robots offered a welcomed social presence during reading; (2) their animations and responses during reading impacted the experience positively by promoting reading engagement, enjoyment, and comprehension, but could also be distracting when unexpected; and (3) reading robots appear to be intelligent, literate and helpful when they have the capacity to talk. Children also highlighted an expectation for corrective feedback and demonstrated relatively accurate understandings about the capabilities and limits of robot intelligence.

### Theme 1. Robots offer a welcomed social presence

Most children expressed that they welcomed, enjoyed and benefited from the robot’s physical social presence, irrespective of the robot selected. This included being comforted, calmed, supported and motivated by the robot’s presence. Some children indicated that they liked their robot because it kept them ‘company’, suggesting that the social presence of the robot alone was engaging. Others indicated that being invigilated by an attentive robot while reading was motivating. These initial findings provide some tentative support for using physically-embodied robot agents as an education tool over screen-based artificial agents which may not be sufficient in generating the social context necessary to motivate and engage children in educational activities such as reading^24–26^.

Critically, the social presence offered by the robot was usually perceived as unintimidating. Some children explicitly touched on the notion that robots can offer a non-judgemental companion mitigating any anxiety about making an error when reading. Other studies examining perceptions towards robots in a motivational interviewing context have similarly reported that some individuals preferred to be interviewed by the NAO robot than a human stranger as they were less likely to feel intimated or judged^27^. However, some variability emerged among children, with three children indicating that they might still experience apprehension and embarrassment at the prospect of making a mistake when reading to their robot. When specifically asked if they thought the robot would or could judge them, children explicitly stated that they did not think this was likely. This reveals the complexity of expectations and perceptions children (and, likely, adults) can have towards artificial agents when they are positioned to demonstrate ‘intelligent’ and human-like capabilities. Specifically, it is possible for an individual to explicitly perceive an agent as artificial, and lacking human-level intelligence, sentience and intentions, and yet, experience visceral or subconscious reactions to the agent as they would a human. This highlights the need to develop tools that can more sensitively probe the explicit and implicit stances humans take towards robot agents in various contexts^28^. Furthermore, in order to confirm the utility of reading robots as a tool to establish non-judgmental, unintimidating social contexts for reading, this work must be extended to children with a history of reading difficulty and reading/social anxiety. Whilst we attempted to explore variability in experiences across individual differences in reading fluency and reading/social anxiety, the variability in our sample was inadequate to draw any meaningful conclusions in this regard. Prospective studies which specifically sample these children are now needed.

### Theme 2. Robot animations can engage and distract

Whilst some children leaned on the robot’s aesthetics to make inferences about its intelligence and reading competence, it was the robot’s animations and reactions during the reading activity that appeared to most significantly shape children’s perceptions. This is consistent with research at the intersection of human—robot interaction and social neuroscience which shows that there are many factors that influence how we perceive, behave and feel when we interact with robots other artificial agents. This can include how they look as well as our beliefs about their intelligence, sentience and capacity for intentional actions^9^.

Our findings show that the robot animations in particular signalled the robot’s capacity to attend to and understand the child and story. Some children went further to say that the robot’s emotive reactions at key plot-points facilitated their reading comprehension. Indeed this aligns with past research which shows that reading comprehension in children benefited from the presence and social interactions of a robot co-reader^29^. These findings are highly encouraging for the future deployment of robots as reading companions; however, it was also clear that current robot capabilities are inadequate for fully realising this potential. Specifically, robot reactions that were even slightly mis-timed or unexpected had the potential to distract children when reading. A Wizard-of-Oz approach was specifically implemented in the current study to minimise such issues, while also ensuring we had maximal control over the timing of robot animations, irrespective of the child’s reading speed and accuracy. Nevertheless, even this approach could not guarantee optimally-timed responses. More importantly, human-controller approaches negate the key advantages that robot companions provide: scalability, availability and non-judgmental privacy, since an adult operator must be nearby.

Indeed, we attempted to automate the robot animations using a speech recognition algorithm (this automated function is available as an option in our robot control application, see Method for links to code). However, this was inadequate because current speech recognition applications are too slow and inaccurate to support real-time, rapid reactions during fluent, let alone, dysfluent, reading. Voice recognition is already known to be challenging in children due to idiosyncratic pitch and speech dysfluency^30^. This challenge is exacerbated when children have reading difficulty and mispronounce words. A further concern was that a mistimed response could have the potential to do more harm than good if misinterpreted by the child. For instance, a delayed response in which the robot laughs at a humorous plot point could be misinterpreted as the robot laughing at the child’s reading. This would be especially problematic for children with reading difficulty who may already experience anxiety and a fear of negative evaluation during reading^16,31^. This exemplifies a much broader challenges of automation in human—robot interaction, especially when interactions involve verbal communication^32–34^. Previous longitudinal observations of in-home interactions with the humanoid robot, Pepper (SoftBank Robotics) have revealed that people expect social robots to autonomously sustain complex reciprocal conversations, and that the inability of robots to do this results in both disappointment and diminished interest/engagement^35^.

### Theme 3. Helpful reading robots can talk

Children expressed that the capacity for verbal communication is a key feature expected of a robot reading companion. For many children the presence or absence of this capability was a marker of the robot’s intelligence. In particular, children often judged a robot’s capacity to read and understand the story based on the robot’s ability to talk. Furthermore, children indicated a desire and expectation for reading robots to provide corrective feedback when needed. This was surprising as the robot was not at all positioned as a teacher or tutor in this reading scenario.

Importantly, however, some children specifically described the way in which this feedback should be offered. One suggested that it could be to prompt the child when an error was made rather than offering an abrupt correction; another believed that feedback from a robot would be less harsh than feedback from a human teacher; and another suggested that it should be up to the child to request assistance when needed, thus affording them more control over when feedback is provided. Recently, there has been growing discussion around what precise social roles robots should play in education settings, with emerging research suggesting that positive learning outcomes are observed when children learn alongside, or even ‘teach’ a robot peer or novice^1^. Our findings suggest that children may benefit from robots who serve mixed roles: in this case, a peer who can follow and co-experience the child’s learning, but also offer help and instruction when requested.

## Limitations

In the current study, all children read the same book to standardise the reading experiences as much as possible – including the quality and quantity of robot animations. Further, an easy text was intentionally selected to minimise reading difficulties for younger children in the sample. A limitation of this approach is that the text is likely to have appeared too easy for some children. This may have limited children’s evaluations of how helpful they thought the robot was, or could be for themselves. Accordingly, the oldest children expressed that the robot would benefit younger children in earlier stages of learning to read. Future work should evaluate children’s experiences with robot reading companions when reading texts are matched, or slightly exceed, the child’s reading ability. This follows in that the application of robots to promote reading engagement is likely to be most impactful when children read books that they find difficult.

It is important to note that the current study only examined experiences following a single and short reading activity. Consequently, the reflections from children assessed here may, in part, reflect the novelty of robots. This is especially likely to be true for children’s reflections on robots being ‘cool’ and ‘fun’ (sub-theme 1.2) and effective in promoting engagement by dynamically reacting to story plot points (sub-theme 2.1). Longitudinal data is needed to determine whether these potential benefits of robots persist over time when children engage with robots in the home or classroom, and indeed, whether children themselves would initiate interactions with robots for the purpose of reading. Previous studies applying social robots for education interventions have highlighted challenges in maintaining children’s engagement with robots^34^. This may be, in part, due to waning novelty and a realisation of the robot’s limited capabilities (e.g., capacity for complex and reciprocal conversation)^35,36^.

Finally, it is important to note that the insights gained from the current study on the application of social robots as reading and learning companions may not generalise to older children and adults since perceptions of robots have been shown to change with development, possibly as the ability and tendency to attribute mental states to others matures. For instance, Brink and colleagues^37^ have shown that younger children (< 9 years) were less likely to experience the ‘uncanny valley effect’ towards human-like robots, and as a result were more open to interactions with them. As such, future work which begins to examine the experiences of children with reading difficulty and anxiety should broadly sample readers or all ages.

### Conclusion

The current study shows much promise for the application of social robots to support children’s reading engagement. Whilst similar benefits have been reported with reading assistant dogs (see Introduction for an in-depth review), robots have the potential to offer a much more practical, economical, controlled and scalable option. Indeed, one key benefit of social robots, over human or animal companions, is that they offer on-demand availability. However, for the full potential of reading robots to be realised, technological strides in developing autonomous robots that have highly sensitive and accurate speech recognition – for both correct and incorrect pronunciations of regular and irregular words – is needed, to ensure they can engage in an appropriate and timely way. This is also key if robots are to additionally provide children with corrective feedback when it is both needed and wanted.

More broadly, we demonstrate that much is to be gained from commencing the design and application of social robotics in education contexts by asking children to share their impressions, perspectives and experiences. This offers the opportunity to identify the robot features that are likely to help and hinder children when learning alongside artificial companions.

## Method

### Participants

Thirty children (*M*_age_ = 8.51 years, *SD* = 1.74; 11 females; 67% Caucasian, 33% Asian) were recruited from the general public through word of mouth and social media advertising in the greater Sydney area. Both children and their parents provided written and informed consent before taking part in the study. This study was conducted in accordance with the protocol approved by the Macquarie University Human Research Ethics Committee.

### Procedure

**Figure 7:**
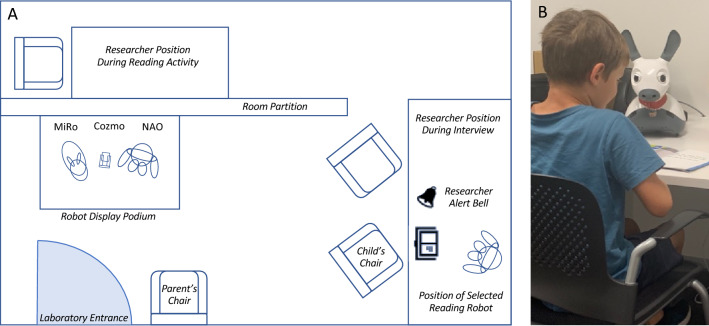
(**A**) Schematic depiction of laboratory layout. (**B**) Photograph of child model reading with a robot. Photo of model depicted used with the informed consent of the child and their parents. This child was not a participant in the current study.

Participants were given the option for parents to sit in on the study session. Only three parents chose this option. All parents were asked to complete a demographic questionnaire, as well as two anxiety measures which assessed their child’s reading and social anxiety (see Measures below). Once information and consent forms were completed, participants were taken to the robotics laboratory where three robots were positioned on a small table by the entrance (see Fig. 7). At this point, the robots were inactive and children were simply told the names of each robot. The researcher and child then sat down beside the robots and the researcher asked a series of questions about the child’s initial impressions of the three robots. Answers to all questions were directly entered into an online survey completed by the researcher on an iPad. See Supplementary Material 2 for a complete depiction of the survey questions and layout. Quantitative survey data and associated analysis code is available on the Open Science Framework (https://osf.io/jdv2y/). Survey data from one participant was lost due to a data upload error, however this individual was still included in the qualitative analyses which used audio recordings of a subsequent interview (see below).

### Phase 1. First impressions and expectations

The initial phase of the interview aimed to assess children’s first impressions of the different robots. In order to explore the extent to which these first impressions were driven by robot aesthetics and function, we first asked children to rank the *inactive* robots in order of preference, and explain their preferences. This was done using a graphical interface on the iPad in which the child could drag and place a 1st place, 2nd place and 3rd place ribbon onto each of the robots. Children were then asked if they have any questions about the robots.

**Figure 8:**
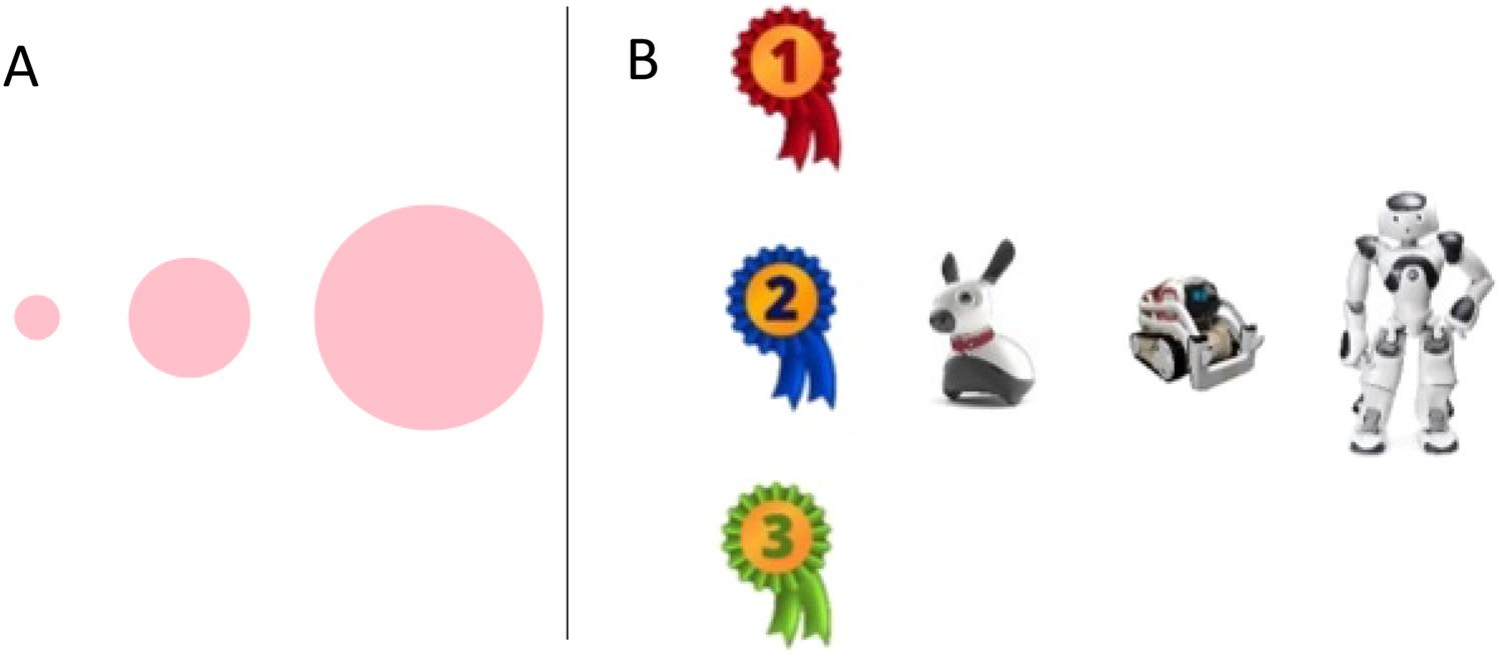
(**A**) Visual rating scale. (**B**) Robot preference ranking display screen.

Then children were shown three short videos which displayed each robot *in action* (see Supplementary Material 3). These videos simply depicted each of the robots rolling a small ball along a platform, moving forwards in the same direction as the ball, and then reacting to the ball dropping off the platform. The purpose of these videos was to highlight the key functional differences between the three robots, such as verbal communication for NAO, tail wagging and light displays for MiRo and emotive expressions for Cozmo. Videos were presented to children in a randomised order. After each video, children were asked if they had any questions about the robot, as well as how “Friendly” and “Intelligent” they thought the robot was. These ratings were made on a visual 3-point scale depicted by three red circles of increasing size (see Fig. 8A). Once all three videos were viewed, the child was asked to rank the robots again according to their preferences using the same drag-and-drop interface (see Fig. 8B).

### Phase 2. Reading with preferred robot

Children were then told that they would now have the opportunity to read a short book with their favourite robot. Before commencing the reading activity, children completed a visual adaptation of the brief State-Trait Anxiety Inventory^38^ to obtain a measure of their state anxiety immediately after being instructed that they would be reading with the robot. Children were asked to indicate how “calm”, “tense”, “upset”, “relaxed”, “content” and “worried” they were using the same visual 3-point scale depicted above (see Fig. 2) and was numerically scored as 1–3 for each item, with a possible range of 6–18.

Children then sat at a table with their chosen robot positioned approximately 45 degrees to their right. The researcher gave all children the same book to read; ‘At the Fun Run’ by Lorraine Lea. This book was selected from the ‘Little Learners Love Literacy®’ story series (www.learninglogic.com.au), and is categorised as a Stage4, level 16. This book was chosen so that it was simple enough for all children in the sample to read it accurately, whilst still being engaging enough to allow for a range of robot reactions and animations. We wanted to use the same book with all participants in order to standardise the robot’s animations during the story.

Once the child was ready to begin reading, the researcher told the child that they would be doing some work on the other side of the laboratory, behind a large room partition that visually occluded the experimenter from the child. The researcher then placed a cowbell on the table and instructed the child: *“I will be wearing my headphones, but when you are done you can get my attention by ringing this bell.”* This instruction was provided to minimise any influence of the researcher’s physical presence in the room. In reality, the researcher was listening to the child behind the partition and controlled the robot’s animations at four pre-determined points in the story using a Wizard of Oz approach. This was achieved using a remote-control application that was built into the online survey (see Supplementary Material 2; code available on GitHub: https://github.com/gimait/readingtorobot_app; https://github.com/chaudhuryB/ReadingToRobot). Animations depicted happiness/excitement; fear/surprise, annoyance or sadness. For example, when the child read “*I cut my leg*” NAO would sympathetically say “Aww!”, Cozmo would make a sad vocalisation accompanied by a downcast expression, and MiRo would tilt its head slowly towards the table. Videos depicting all animation reactions by robot are available on the Open Science Framework (https://osf.io/jdv2y/) and a detailed guide on animation development for each animation and robot can be found in the design documentation on the associated GitHub page (https://github.com/chaudhuryB/ReadingToRobot/blob/master/doc/design.md). Once the child completed the story and rang the bell, the researcher returned and re-administered the same state anxiety items using the iPad interface.

### Phase 3. Structured interview to probe reading experience

The researcher then conducted a structured interview which comprised a combination of subjective rating questions, alternative forced choice questions and open-ended questions (see Supplementary Material 2 for a complete list of questions). These questions probed the aspects of the robots, and reading with them that children liked and disliked, as well as the extent to which they found the robot *helpful, a good listener, a good teacher,* and *kind.* We also asked open-ended questions to explore robot features that would make the robot a better reading companion and to characterise children’s perspectives about how robots work, and how they should be used (e.g., *“What types of activities could you do with this robot?”*).

### Qualitative thematic analysis of interview data

This interview was audio-recorded with participants’ consent, except for one child who did not provide consent. For this child, responses were recorded verbatim in writing during the interview with their consent. All responses were transcribed and analysed by N.C. and R.M. using a reflexive thematic analysis approach^39,40^. The goal of this analysis was to accurately capture the experiences and perceptions of children when engaging with the robots during reading. N.C. and R.M. began discussing trends in children’s experiences throughout the data collection process and identified potential codes that might be relevant for thematic analysis. Once all interviews had been transcribed, N.C. immersed themselves in the data, reading all the transcripts twice and applying relevant codes to each using NVivo (Version 12). N.C. then organised codes under unifying themes. This initial thematic analysis was then discussed with both R.M. and E.C., who also reviewed all coded transcripts (see Supplementary Material 4 for deidentified transcripts). This ultimately resulted in some codes being consolidated into common codes or separated into more specific codes. The final coding and themes were then agreed upon by all authors, consistent with Braun and Clarke’s inductive and iterative method for thematic analysis.

## Measures

We administered screening tests for reading and anxiety to characterise our sample and contextualise their reflections following the reading activity. See Table 2 for a full summary of participant scores on these measures.

**Table 2:** Screening measures of reading efficiency and anxiety.

Measures	M	SD
TOWRE – sight word reading	103.97	17.24
TOWRE – phonemic decoding	101.63	11.72
MORAT-P – reading anxiety	6.77	5.13
SPENCE-P total	17.40	13.07
SPENCE-P – social anxiety	3.27	2.83

Standard scores are reported for both TOWRE subtests. Raw scores are reported for the MORAT-P and SPENCE-P social anxiety subscale.

### Test of word reading efficiency 2 (TOWRE 2)

Word reading efficiency was assessed in all children using Form A from both subtests of the TOWRE 2^41,42^. The Sight Word subtest requires participants to read a list of regular and irregular words as quickly and as accurately as possible in 45 s. This test provides a measure of how efficiently children can read sight words by recognizing their orthographic representation. The Phonemic Decoding subtest is administered in the same way but involves reading a list of ‘non-words’. This test provides a measure of how efficiently children can read words using letter-sound rules. The list in both subtests is ordered so that items are of increasing difficulty. The number of correctly pronounced words read in 45 s is summed and then converted into a standard score using available norms.

### Macquarie oxford reading anxiety test – parent report (MORAT-P)

The parent report version of the MORAT was used to obtain a measure of children’s reading anxiety^43^. The measure comprises 48 statements which describe concerns children may have about reading. Parents are required to rate how often their child experiences these signs of reading anxiety (0 = “never” / “I don’t understand”, 1 = “sometimes”, 2 = “often”, 3 = “always”). Total raw scores are reported and included in analyses.

### Spence children’s anxiety scale – parent report (SPENCE-P)

The parent report version of the SPENCE was used to obtain an overall measure of anxiety, as well as a specific measure of social phobia^44^. The measure comprises of 38 items which can be divided into six subscales (generalized anxiety; separation anxiety; social anxiety; panic/agoraphobia; physical injury fears; obsessive–compulsive symptoms). The current study was particularly interested in the social anxiety subscale. Each item describes an anxiety symptom and parents rate the frequency this is experienced by their child (0 = never, 1 = sometimes, 2 = often, 3 = always). These subscales have been reported to have sound internal consistency, criterion validity and test–retest reliability^45^.

## Data Availability

The datasets generated and analysed during the current study are available in the Open Science Framework repository, along with associated code and other supplementary materials (https://osf.io/jdv2y/).

## References

[1] Belpaeme T, Kennedy J, Ramachandran A, Scassellati B, Tanaka F (2018). Social robots for education: A review. Sci. Robot..

[2] Papakostas GA (2021). Social robots in special education: A systematic review. Electronics.

[3] Caruana N, de Lissa P, McArthur G (2017). Beliefs about human agency influence the neural processing of gaze during joint attention. Soc. Neurosci..

[4] Caruana N, Spirou D, Brock J (2017). Human agency beliefs influence behaviour during virtual social interactions. PeerJ.

[5] Caruana N, McArthur G (2019). The mind minds minds: The effect of intentional stance on the neural encoding of joint attention. Cogn. Affect. Behav. Neurosci..

[6] Cross, E. S., Ramsey, R., Liepelt, R., Prinz, W. & Hamilton, A. F. de C. The shaping of social perception by stimulus and knowledge cues to human animacy. *Philos. Trans. R. Soc. B Biol. Sci.* **371**, 20150075 (2016). 10.1098/rstb.2015.0075 PMC4685521 26644594

[7] Cross, E. S., Hortensius, R. & Wykowska, A. From social brains to social robots: applying neurocognitive insights to human–robot interaction. *Philos. Trans. R. Soc. B Biol. Sci.* **374**, 20180024 (2019). 10.1098/rstb.2018.0024 PMC6452245 30852997

[8] Hortensius R, Cross ES (2018). From automata to animate beings: the scope and limits of attributing socialness to artificial agents: Socialness attribution and artificial agents. Ann. N. Y. Acad. Sci..

[9] Cross ES, Ramsey R (2021). Mind Meets Machine: Towards a Cognitive Science of Human-Machine Interactions. Trends Cogn. Sci..

[10] Bamkin M, Goulding A, Maynard S (2013). The children sat and listened: Storytelling on children’s mobile libraries. New Rev. Child. Lit. Librariansh..

[11] Fridin M (2014). Storytelling by a kindergarten social assistive robot: A tool for constructive learning in preschool education. Comput. Educ..

[12] McArthur, G. *et al.* Phonics training for English-speaking poor readers. *Cochrane Database Syst. Rev.* **2018**, (2018). 10.1002/14651858.CD009115.pub3 PMC6517252 30480759

[13] McArthur G, Castles A (2017). Helping children with reading difficulties: Some things we have learned so far. Npj Sci. Learn..

[14] Michaelis, J. E. & Mutlu, B. Reading socially: Transforming the in-home reading experience with a learning-companion robot. *Sci. Robot.* **3**, eaat5999 (2018). 10.1126/scirobotics.aat5999 33141721

[15] Rohlfing KJ (2022). Social/dialogical roles of social robots in supporting children’s learning of language and literacy—A review and analysis of innovative roles. Front. Robot. AI.

[16] Francis DA, Caruana N, Hudson JL, McArthur GM (2019). The association between poor reading and internalising problems: A systematic review and meta-analysis. Clin. Psychol. Rev..

[17] McArthur, G., Badcock, N., Castles, A. & Robidoux, S. Tracking the relations between children’s reading and emotional health across time: Evidence from four large longitudinal studies. *Read. Res. Q.* **n/a**, (2021).

[18] Jalongo MR, Hirsh RA (2010). Understanding reading anxiety: New insights from neuroscience. Early Child. Educ. J..

[19] Francis D, Hudson JL, Kohnen S, Mobach L, McArthur GM (2021). The effect of an integrated reading and anxiety intervention for poor readers with anxiety. PeerJ.

[20] Clark, C. & Douglas, J. *Young People’s Reading and Writing: An In-Depth Study Focusing on Enjoyment, Behaviour, Attitudes and Attainment*. *National Literacy Trust* (National Literacy Trust, 2011).

[21] Hall SS, Gee NR, Mills DS (2016). Children reading to dogs: A systematic review of the literature. PLoS ONE.

[22] Burdett ERR, Ikari S, Nakawake Y (2022). British children’s and adults’ perceptions of robots. Hum. Behav. Emerg. Technol..

[23] Lupetti, M. Robots, Aesthetics, and the heritage context. *Assoc. Comput. Mach.* **XXIV**, 6 (2017).

[24] Henschel A, Hortensius R, Cross ES (2020). Social cognition in the age of human-robot interaction. Trends Neurosci..

[25] Hortensius R, Hekele F, Cross ES (2018). The perception of emotion in artificial agents. IEEE Trans. Cogn. Dev. Syst..

[26] Laban G, George J-N, Morrison V, Cross ES (2020). Tell me more! Assessing interactions with social robots from speech. Paladyn J. Behav. Robot..

[27] Galvão Gomes da Silva, J. *et al.* Experiences of a motivational interview delivered by a robot: Qualitative study. *J. Med. Internet Res.* **20**, e116 (2018). 10.2196/jmir.7737 PMC5958282 29724701

[28] Marchesi, S., Spatola, N., Perez-Osorio, J. & Wykowska, A. Human vs humanoid. A behavioral investigation of the individual tendency to adopt the intentional stance. in *Proceedings of the 2021 ACM/IEEE International Conference on Human-Robot Interaction* 332–340 (ACM, 2021). doi:10.1145/3434073.3444663.

[29] Yueh H, Lin W, Wang S, Fu L (2020). Reading with robot and human companions in library literacy activities: A comparison study. Br. J. Educ. Technol..

[30] Kennedy, J. *et al.* Child speech recognition in human-robot interaction: Evaluations and recommendations. in *Proceedings of the 2017 ACM/IEEE International Conference on Human-Robot Interaction* 82–90 (ACM, 2017). doi:10.1145/2909824.3020229.

[31] McArthur GM, Filardi N, Francis DA, Boyes ME, Badcock NA (2020). Self-concept in poor readers: A systematic review and meta-analysis. PeerJ.

[32] Henschel A, Laban G, Cross ES (2021). What makes a robot social? A review of social robots from science fiction to a home or hospital near you. Curr. Robot. Rep..

[33] Kanda T, Hirano T, Eaton D, Ishiguro H (2004). Interactive robots as social partners and peer tutors for children: A field trial. Hum. Comput. Interact..

[34] Leite I, Martinho C, Paiva A (2013). Social robots for long-term interaction: A survey. Int. J. Soc. Robot..

[35] Dereshev, D., Kirk, D., Matsumura, K. & Maeda, T. Long-term value of social robots through the eyes of expert users. in *Proceedings of the 2019 CHI Conference on Human Factors in Computing Systems* 1–12 (ACM, 2019). doi:10.1145/3290605.3300896.

[36] Tanaka, F. *et al.* Pepper learns together with children: Development of an educational application. in *2015 IEEE-RAS 15th International Conference on Humanoid Robots (Humanoids)* 270–275 (IEEE, 2015). doi:10.1109/HUMANOIDS.2015.7363546.

[37] Brink KA, Gray K, Wellman HM (2019). Creepiness creeps. Uncanny valley feelings are acquired in childhood. Child Dev..

[38] Marteau TM, Bekker H (1992). The development of a six-item short-form of the state scale of the Spielberger State-Trait Anxiety Inventory (STAI). Br. J. Clin. Psychol..

[39] Braun V, Clarke V (2006). Using thematic analysis in psychology. Qual. Res. Psychol..

[40] Braun V, Clarke V (2019). Reflecting on reflexive thematic analysis. Qual. Res. Sport Exerc. Health.

[41] Tarar, J. M., Meisinger, E. B. & Dickens, R. H. *Test review: Test of word reading efficiency–second edition (TOWRE-2)* by Torgesen, J. K., Wagner, R. K., & Rashotte, C. A. *Can. J. Sch. Psychol.* **30**, 320–326 (2015).

[42] Torgesen, J. K., Wagner, R. K. & Rashotte, C. A. *Test of Word Reading Efficiency - Second Edition*. (2012).

[43] Francis, D., Nation, K. & McArthur, G. *The Macquarie Oxford Reading Anxiety Test-Adolescents (MoRAT-Ad)*. (2020).

[44] Nauta MH (2004). A parent-report measure of children’s anxiety: Psychometric properties and comparison with child-report in a clinic and normal sample. Behav. Res. Ther..

[45] Orgilés M, Rodríguez-Menchón M, Fernández-Martínez I, Morales A, Espada JP (2019). Validation of the parent report version of the Spence Children’s Anxiety Scale (SCAS-P) for Spanish children. Clin. Child Psychol. Psychiatry.

